# Comprehensive Characterisation of Extracellular Vesicle Preparations Using Multiparametric Size-Exclusion Chromatography

**DOI:** 10.3390/ijms26178477

**Published:** 2025-08-31

**Authors:** Darja Božič, Katja Vrabec, Ana Železnik, Andrej Raspor, Valentina Novak, Ivana Petrović Koshmak, Maja Leskovec, Aleš Štrancar

**Affiliations:** Sartorius Bia Separations, Mirce 21, SI-5270 Ajdovščina, Slovenia

**Keywords:** extracellular vesicles, analytical chromatography, fluorescence, MALS, PAT, immunolabelling, quality control

## Abstract

Extracellular vesicles (EVs) hold great promise in the fields of diagnostics and therapeutics. However, the heterogeneity of these membrane-enclosed messengers and the complexity of the biological samples in which they occur pose significant research challenges. The aim of this study was to improve the reliability of size-exclusion chromatography (SEC) and immunolabelling as common approaches in the EV field, and to provide a comprehensive characterisation tool for diverse EV preparations. Profiling of SEC-separated sample components was conducted through light absorbance, fluorescence, and light scattering, providing insights into particle content, size, protein content, and specific markers. Key considerations for assay robustness, including SEC mobile phase composition and immunostaining parameters, were addressed. Respecting the importance of controlled immunolabelling and preanalytical factors, the method efficiently reveals changes in the sample profile with respect to particles and small impurities. The detailed analytical capabilities and method adaptability offer a practical way to enhance the efficiency of EV research and applications.

## 1. Introduction

Extracellular vesicles (EVs) are one of the latest biological nanoparticles to be recognised as important mediators of intercellular transport and modulation [1]. The heterogeneity of EVs themselves, coupled with the complexity of biological samples that often contain other biological assemblies with similar or overlapping physicochemical properties (e.g., lipoproteins, protein complexes, viruses, etc., with no less important functions), still represents a substantial analytical challenge. Despite significant technological advancements, reliable, fast, and robust methods are still lacking [2].

Rigorous characterisation of EV preparations usually consists of several complementary techniques. Traditional methods include nanoparticle tracking analysis (NTA), dynamic light scattering, tunable resistive pulse sensing (TRPS), flow cytometry (FCM), and electron microscopy, providing foundational insights into particle size distribution, concentration, surface markers, and structure [3]. Additional methods are typically applied to quantify the contents of proteins, nucleic acids, and lipids, and confirm the presence of selected EV markers [2,3]. SEC is a powerful tool allowing for the separation and characterisation of various biological compounds, and in the field of EVs, SEC is one of the most widely used methods for EV isolation. However, few publications can be found employing SEC as a characterisation tool for EV samples [4,5,6,7,8], and SEC as an analytical approach is absent in most recent reviews of methods for EV characterisation [3,9,10,11]. Our aim was to establish a robust method that would allow for a comprehensive characterisation of EV samples with varying complexity and concentration. Analytical SEC was coupled with absorbance, fluorescence, and light scattering detectors to extract information on particle content, particle size, protein content, and EV surface markers (CD9/CD81/CD63). Furthermore, we addressed the impacts of mobile phase composition and critical parameters for reproducible immunolabelling. Nanoparticle tracking analysis and Bradford assay were used to support the interpretation of the parameters extracted from SEC.

In this article, we share an approach that provides fundamental insights into sample composition at a reasonable throughput (the method requires 30 min per injection and can be shortened down to 18 min for purified samples), which requires minimal prior knowledge about the sample and sample preparation. Although SEC-based characterisation has been previously discussed in the EV literature, to the best of our knowledge, this study is the first to provide a detailed protocol along with a critical evaluation of its applicability. Further validation within the context of a specific study is beyond the scope of this work, but several relevant tests demonstrating the reproducibility and robustness of the method are provided. We believe that the possibility of streamlining analytics in the early stages of research will be of great interest to researchers in the field.

## 2. Results

### 2.1. SEC Analysis and Characterisation Strategy

**Figure 1:**
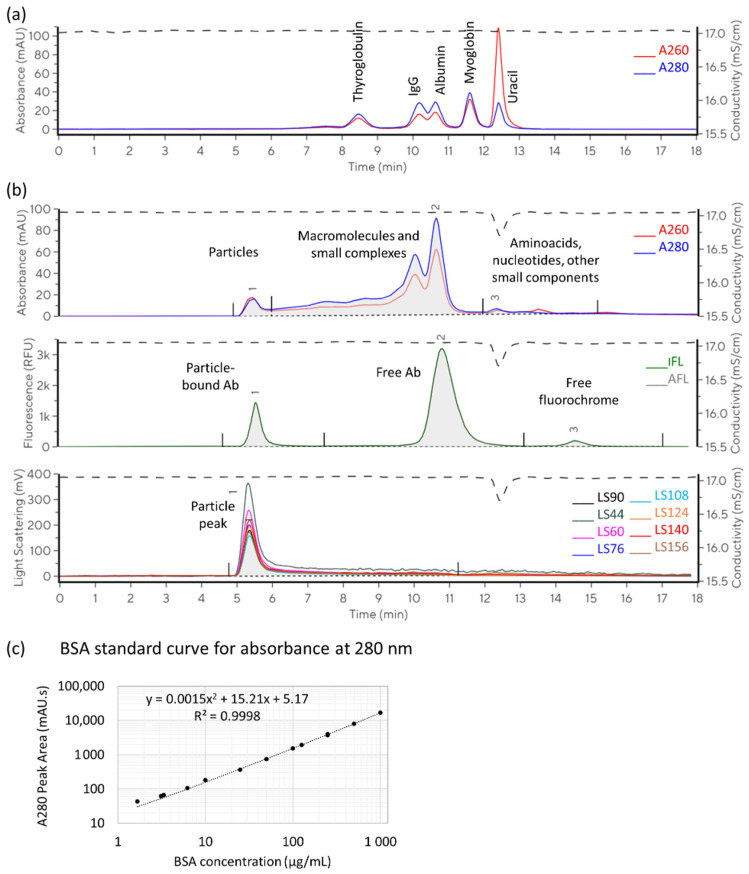
SEC chromatogram evaluation strategy. A260—light absorbance at 260 nm, A280—light absorbance at 280 nm, IFL—immunofluorescence, AFL—autofluorescence, and LS—light scattering. (**a**) Chromatogram of 5-component standard (BEH200 SEC Test Mix). Peaks 1, 2, and 3 were considered in running buffer optimisation. (**b**) An overlay of representative chromatograms obtained for non-labelled and immunolabelled TFF retentate samples. Absorbance, autofluorescence, and light scattering signals are shown for the non-labelled sample, and immunofluorescence is shown for the CD63-FITC antibody. The integrated area of absorbance at 280 nm is divided into particle-related (1), macromolecule-related (2), and small molecule-related (3) peaks. The integrated area of fluorescence is divided into particle-bound (1), free antibody (2), and free fluorochrome (3) peaks. Multi-angle light scattering was used for the calculation of particle size, while the signal measured at 90° (LS90) was evaluated as a measure of particle concentration and for visual comparison of different sample profiles. (**c**) Standard curve for A280 response (integrated peak area) as a function of protein concentration in the sample. The results were obtained using bovine serum albumin (BSA).

SEC chromatograms can provide an immense amount of information. We focused on the characterisation and quantification of particles in the tested samples; however, the analysis could also be upgraded for profiling and quantification of non-particle-associated components. Figure 1 illustrates the evaluation approach and main parameters assessed in this study.

In the described method setup, particle components exceeding the size of about 30 nm elute close to the column void volume, at retention times of 4.5–6 min. Smaller components elute later. The separation of proteins of different sizes and uracil is shown in Figure 1a for orientation regarding the relation between the size and retention time. Dual wavelength absorbance monitoring, with a characteristic A260/A280 ratio, was employed for tracking peaks related to proteins and nucleic acids. The integrated area of absorbance measured at 280 nm (A280) was used to evaluate protein content, while multi-angle light scattering (MALS) was used for estimating particle size. With respect to the preserved particle size, the integrated area of light scattering measured at a 90° angle (LS90) served as a measure of particle concentration and for visual comparison of different sample profiles. Changes in peak shape and retention time were monitored as indicators of changes in particle population and/or integrity.

**Figure 2:**
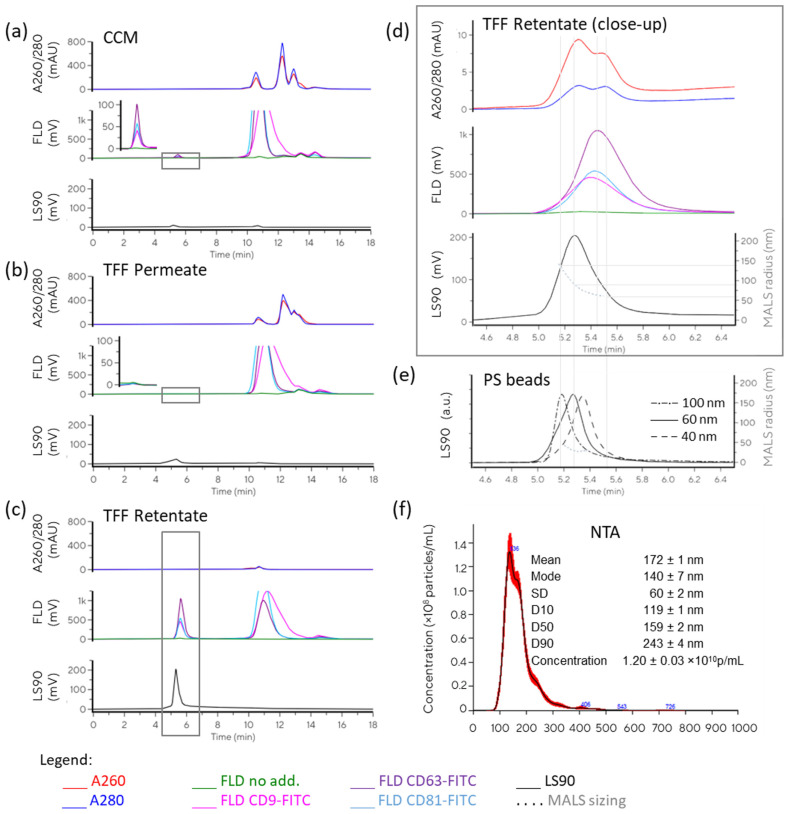
Characterisation of EV preparation. (**a**) Overlay of chromatograms obtained in SEC analysis of clarified conditioned medium (CCM), with and without antibody probes against CD9, CD63, and CD81; the black frame marks the section of chromatogram presented in a close-up; (**b**) overlay of chromatograms obtained in SEC analysis of TFF permeate, with and without antibodies against CD9, CD63, and CD81; the black frame marks the section of chromatogram presented in a close-up; (**c**) overlay of chromatograms obtained in SEC analysis of TFF retentate (EV-enriched sample), with and without antibody probes against CD9, CD63, and CD81; the black frame marks the section of chromatogram presented in (**d**) close-up of the particle peak of TFF retentate; and (**e**) overlay of chromatograms obtained in SEC analysis of polystyrene size standards with nominal diameters of 100, 60, and 40 nm, respectively; only LS90° signals are shown for this sample. The MALS radius calculation (grey dotted line) is shown only for 60 nm beads. (**f**) The result of NTA of TFF retentate.

The chromatograms of clarified conditioned media (CCM), TFF permeate, and TFF retentate (Figure 2a–c) clearly illustrate the changes in the sample during TFF processing. It is evident that the permeate contained the majority of small components from the CCM, while no particles were detected through MALS and EV markers in that sample. Conversely, TFF retentate was largely depleted of small components, and enrichment in particles was indicated by increased LS and EV marker-related fluorescence.

Based on the BSA calibration curve, Figure 1c shows the integrated area of protein-related components (A280-prevalent peaks with retention time shorter than 12 min, see Figure 2b) that was used to calculate protein contents.

The results of sample characterisation for two exemplary purification processes (tangential flow filtration, TFF) are presented in Table 1. The SEC- and Bradford-derived estimations of protein removal in TFF were 95% and 96% for Process 1, and 79% and 82% for Process 2, respectively (Table 1).

**Figure A1:**
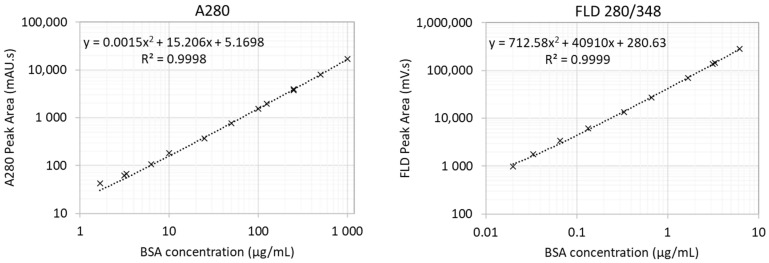
BSA standard curves.

Somewhat larger discrepancies between the results of the two techniques were observed in samples of permeates. Considering the differences in amino acid detection of the two techniques (whereas the A280 method is sensitive to the presence of tryptophan, tyrosine, and phenylalanine [12], the Bradford assay primarily detects arginine and to a lesser extent to histidine, lysine, and aromatic residues [13]) and the different expected interferences in the two approaches, we find these results to be well aligned. In samples with much lower protein contents, tryptophan fluorescence can be applied for protein quantification in SEC analytics (the standard curve for BSA is provided in the Appendix A Figure A1).

We further assessed immunofluorescence. Autofluorescence, antibody aggregation, and non-specific antibody binding were excluded with suitable control samples. The proportions between the EV markers were largely consistent in CCM and TFF RET. In TFF Process 1, the calculated enrichment factors of tetraspanin markers in retentate were markedly larger than the enrichment factor for total particles (11.4, 11.5, and 10.2 for CD63, CD9, and CD81, respectively, versus 5-fold enrichment of particle count based on NTA), while in Process 2, the enrichment factors were much more correlated (24.3, 26.5, and 29.2 enrichment for CD63, CD9, and CD81, respectively, versus 26-fold enrichment in total particles as determined using NTA, see Table 1).

MALS was used to evaluate particle size along Peak 1 (Figure 2d,e). While direct comparison of the retention time of the peak in the EV sample to the retention times of polystyrene size standards (Figure 2e) would suggest an average size of about 60 nm, MALS analysis indicated somewhat larger diameters ranging from 120 to 240 nm (170 nm at peak apex, derived using Zimm fit, Figure 2d, Table 1). These values were in accordance with NTA (Figure 2f and Table 1). Although the SEC primarily separates components according to their size, retention times can be slightly shifted due to weak interactions of the analytes with the column’s solid phase [14,15]. Such interactions can be reduced with a suitable mobile phase. For complex samples, complete prevention of secondary interactions might not be achievable without impairing sample integrity. In that case, the optimal conditions represent a compromise between the minimisation of column–sample interactions and preservation of interactions within the sample. The impacts of the mobile phase parameters on analysis results are discussed in Section 2.2.2.

If we compare the particle evaluation of NTA and SEC-MALS, similar particle enrichment parameters were suggested through particle concentration (NTA) and scattering intensity (SEC-LS90°) for the two evaluated TFF processes (Table 1). In addition, sizing values were surprisingly well aligned. One should bear in mind that the two techniques yield different size parameters. While the hydrodynamic radius is assessed using NTA, the radius of gyration is calculated from MALS, and the resulting size distribution is number-weighted in NTA and intensity-weighted in MALS. In our case, the low and high size values in the determinable range of MALS largely coincided with D10 and D90 as obtained from NTA, while the diameter obtained at the LS90 peak apex, in general, was close to the *mean* value as determined using NTA.

As illustrated in the close-up chromatogram in Figure 2d, the discrepancies between the different signals indicated the heterogeneity in the particle population. The ratio between the signals can be assessed to evaluate sample quality. However, the specificities of the physical background of the measured parameters must be considered in interpretation. The light absorbance signals associated with particle peaks may be distorted due to light scattering interferences [16], affecting the accuracy of the measurements. The LS signal is expected to bend towards larger particles (shorter retention times), as scattering intensity increases exponentially with particle size (a 6th power relation with particle radii is expected according to the Rayleigh approximation [17]). On the other hand, the immunofluorescence signal reflects the amount of surface marker molecules, and in the case that all particles carry the same set of membrane components, the peak is expected to be proportionally bent towards higher concentrations of smaller particles due to the increasing surface/volume ratio for smaller particles, and thus towards longer retention times compared with the LS signal. Clearly, the limits of detection of different sensors for different sample components differ, and the set of measured parameters should be selected based on characteristics of interest. In addition, there is an optimal scattering intensity range within which sizing is possible, providing sufficient signal intensity, good signal/noise ratio, and minimal multiple scattering events. The concentration of particles providing this optimal intensity varies with particle size and refractive index.

### 2.2. Robustness of Analysis

#### 2.2.1. Assuring Reproducible Immunolabelling and Quantitation

**Figure 3:**
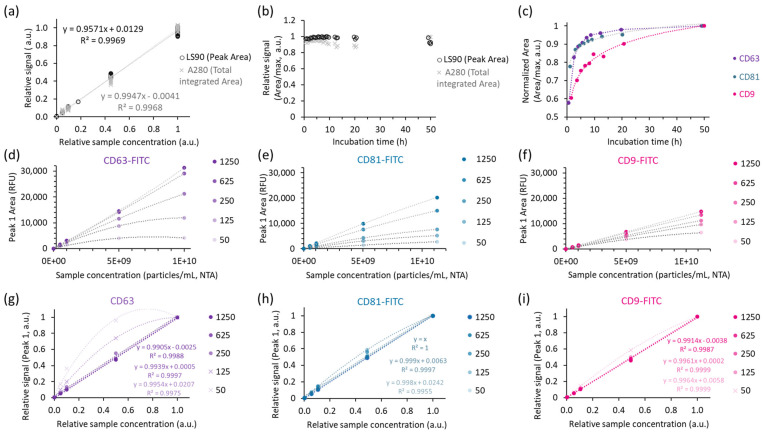
Impact of incubation time and probe concentration on SEC results. Relative values pertain to the ratio between a selected value and the maximal value within the sample series. (**a**) Linear correspondence of sample concentration and integrated area for the LS90 particle peak and total A280. (**b**) Relative A280 and LS90 particle peak area in relation to incubation time. (**c**) Particle peak-related fluorescence with respect to the incubation time (antibody concentration used for immunolabelling was 625 ng/mL). (**d**–**f**) The results of titration experiments: fluorescence Peak 1 area in relation to sample concentration. (**g**–**i**) The results of titration experiments expressed in relative units: relative signal in response to relative sample concentration. The colour scale denotes the antibody concentration used for immunolabelling (50–1250 ng/mL). Data points with evident lack of antibody probe (less than 50% excess in the highest sample concentration) are marked by × signs.

Figure 3 shows the results of sample and probe titration (Figure 3a,d–i) and immunolabelling kinetics at 4°C for the three antibody probes included in this study (Figure 3b,c).

Particle peak (Peak 1)-related LS90 and total A280 signals showed good linear correspondence with the dilution factor within the tested range (Figure 3a) and provided consistent responses for the samples analysed over 2 days of incubation (Figure 3b). The relative standard deviations for the measurements in the three series of samples incubated with different antibody probes for varying times were 6% for A280 (probe contribution to the total A280 was subtracted) and 2% for Peak 1 LS90. The immunolabelling kinetics were different for the three antibodies (Figure 3c), highlighting the importance of incubation time control for a stable immunolabelling assay readout. Considering only the samples with over 8 h of incubation, the relative standard deviations of particle-associated immunofluorescence in this experiment were 2% (CD63), 3% (CD81), and 9% (CD9). Notably, CD9 exhibited the slowest equilibrium establishment, indicating that more than a day of incubation would be required to decrease the variance for that probe. The time of incubation can be shortened using higher concentrations of probes and/or incubation at higher temperatures. However, in this study, incubation at 37 °C was avoided due to increased sample evaporation during incubation at higher temperatures and indications of impaired sample integrity. By applying 1 h of preincubation at 22 °C with mixing, followed by further incubation at 4 °C in the autosampler for at least 8 h, we were able to limit variations in immunostaining of samples throughout the sequence.

By assuring sufficient incubation time, a good linearity and suitable increment in the immunofluorescence were shown in the titration experiment for the three tested probes (Figure 3d–i). These results suggested that a binding equilibrium was established for all three probes, but labelling saturation was only indicated in the case of CD63. The peak area for the other two probes (CD9 and CD81) increased further with higher probe concentration. Considering that in the time dependency experiment, the probe excess was even higher for the latter (Peak 1% FLD Area was 2% for CD9, 3% for CD81, and 5% for CD63), these results suggest a lower affinity for the used CD81 and CD9 antibodies. It is speculated that the poorer accessibility of the binding sites could additionally contribute to the longer time to equilibrium for these two probes (Figure 3c).

#### 2.2.2. Impacts of Running Buffer Composition

In the context of mobile phase composition, we considered the expected impacts of pH, buffer type, and salt concentration. Additionally, the role of poloxamer, which is frequently used as a stabiliser of proteins and cells [18,19,20] but also applied previously in the context of EVs [21,22], was tested. In the current study, we inspected the following parameters: (i) the retention time; (ii) the resolution between the SEC protein standard peaks and between the bound and unbound antibody probes; (iii) the intensity of signals and the ratio between the bound and unbound probes; and (iv) the peak shape parameters (height, width, symmetry, and tailing). The complete dataset on evaluated parameters can be found in Supporting information, Table S1.

Generally, higher salt concentrations and lower pH are expected to increase resolution and reduce the chance of peak tailing [23] on silica-based columns, such as those used in this study. Lower pH reduces the potential ionisation of silanol groups and thus limits the possibility of secondary interactions with the sample components. Furthermore, salt is added to shield the charge of the solid phase and sample components and prevent unspecific adsorption. This is especially important when using a mobile phase with a pH below or close to the isoelectric point of the sample components. The correlation matrix for selected factors and responses analysed in the protein standard (SEC test mix) is presented in Table 2. The protein mix analysis was focused on the elution times of the largest protein, Thyroglobulin (Peak 1, see Figure 1a), and the resolution between the two proteins eluting most closely, namely IgG and albumin (Peaks 2–3, see Figure 1a).

**Figure 4:**
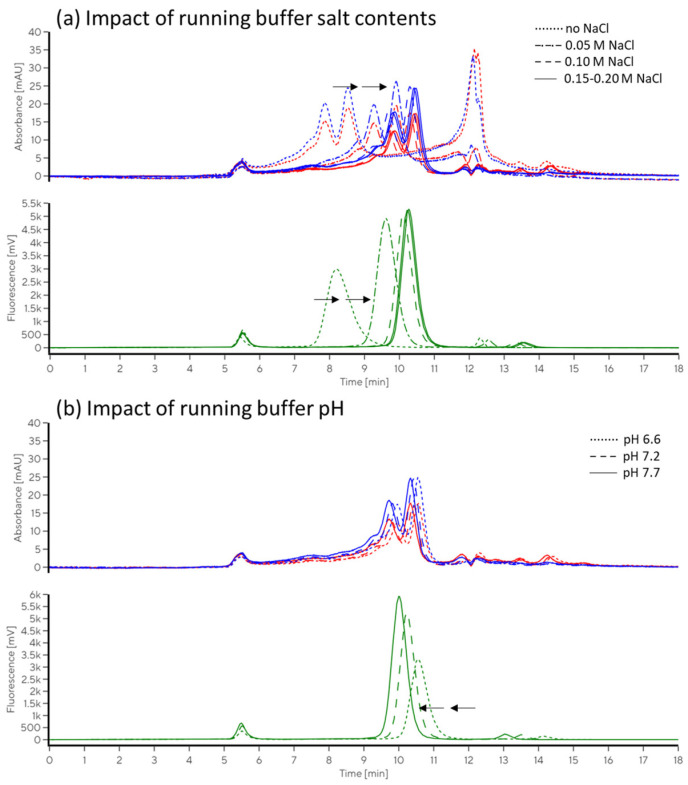
Impact of running buffer composition on SEC separation of CD63 immunolabelled TFF retentate. (**a**) Overlay of chromatograms obtained using a mobile phase with varying salt concentration. (**b**) Overlay of chromatograms obtained for TFF retentate using a mobile phase with varying pH values. All buffers contained 25 mM HEPES. Absorbance at 260 nm (red) and 280 nm (blue) and FITC-conjugated CD63 antibody-related fluorescence (green) are shown.

In the EV sample (CD63-labelled TFF RET), we focused on particle peak parameters (Peak 1, Figure 1b). Additionally, total fluorescence and its distribution between particle-bound probe, free probe, and free fluorochrome (Peaks 1, 2, and 3, respectively, Figure 1b) were followed with the aim of optimising SEC conditions for the evaluation of the immunolabelled sample. The profile of the samples was similar in different buffers, allowing for good separation of bound and unbound antibody probes. Accounting for expected impacts of electrolyte and pH on retention times and resolution of the separation, we perceived no drastic change in the number of peaks or proportions between them that may indicate sample degradation in any of the tested conditions. No significant impact or trend was observed in relation to the addition of poloxamer. The effects of pH and salt variation on resulting chromatograms are illustrated with representative examples in Figure 4. An extract from the correlation analysis is presented in Table 3, and the complete correlation matrix is provided in Supporting information, Table S2.

Table 2 and the EV sample (Table 3). At values below 6.5, pH prevailed as the main factor in the separation of the protein sample, with improved resolution by decreasing pH, while conversely, improved resolution and decreased peak tailing were observed at higher pH values for the EV sample. The impact of increasing salt to improve the separation declined at concentrations above 100 mM NaCl for both samples (the EV sample and protein standard). In the EV sample, the particle peak-related LS90 slowly decreased towards lower pH values, similar to what was previously described in another study [24]. Weak correlations of LS90 Peak 1 height were observed with salt concentration (0.57) and pH (0.44), with reduced peak asymmetry and tailing at higher pH values (−0.53).

Mobile phase composition can strongly impact signal intensities. In this study, the fluorochrome used for immunolabelling was FITC, which is known to be strongly pH-dependent [25]. As expected, higher peaks and larger overall fluorescence were obtained at higher pH values (correlation coefficient 0.98), resulting in almost 20-fold variation in the pH range of 5–8. Increased peak heights were associated with decreased peak width (W05) and tailing (symmetry/tailing coefficients are presented in Table 3, S/T). The separation of the particle peak from the unbound antibody was fine at any of the tested conditions with sufficient salt concentration (at least 20 mM NaCl). Within a sequence, analysed with the same buffer stock and at well-controlled conditions, this FITC fluorescence variance may be of low importance. However, disregarding this effect may lead to substantial inconsistencies, e.g., between the samples analysed in different runs in the case of alterations in buffer preparation or variations in ambient temperature in combination with using temperature-sensitive buffers. Therefore, it is advised to select fluorochromes with more stable fluorescence when possible. In the present case, considering increased FITC fluorescence and improved separation parameters, a slightly higher pH may improve the limit of detection.

At pH values above 7.5, we noticed slight trends of increasing proportions of particle peak (Peak 1)-related fluorescence (bound antibody probe) and LS90 area, and decreasing peak symmetry for both signals. At pH values below 7, there was a trend of increase in Peak 3 (free fluorochrome) % fluorescence. The latter was ascribed to the pH increase at the Peak 3 retention time (the sample was resuspended in dPBS, pH 7.4). Probe degradation and major reduction in probe binding were excluded with a test of incubation of probe alone and with the sample in different buffers (see Section 2.2.3). The change in fluorescence was not associated with a change in the proportion of the fluorescence linked to the particle peak (corr. coef. 0.23) or to the free antibody (corr. coef. 0.26), and only slightly with lower particle peak LS90° at lower pH (0.31), suggesting that the running buffers within the tested range did not substantially impact the sample integrity or stability of the EV–probe complex. However, in the case of using pH-dependent fluorochromes, such as FITC, tight control over the pH is essential to ensure comparability of the measured fluorescence intensities of samples analysed in different sequences. This can be achieved using strong, temperature-insensitive buffers. Comparison of relative values (proportion of total fluorescence related to the particle peak) may be tricky, due to the potential influences of the sample matrices on the unbound probe peaks, with retention time approaching that of salt and other small components.

Despite the clearly pronounced effects of the evaluated factors, we were unable to build a model that would suitably describe all the observed variances in responses. This indicates the complex connections between them and/or the substantial role of additional factors that were not followed in the scope of the present study.

#### 2.2.3. Evaluation of Sample Changes After Exposure to Different Conditions and Immunolabelling Efficiency

To test the sample matrix effect on SEC evaluation, we spiked the TFF RET (from Process 2) into buffer solution with varying pH values (6.5–8) and salt concentration (0.05–1 M NaCl) prior to adding antibody probes. These samples were compared with the sample that was diluted in dPBS. Four parallel samples were prepared for every condition: one aliquot without any addition and one with each of the three antibody probes. All samples were analysed using the same SEC running buffer, following at least 12 h preincubation in the selected buffer and with the selected probe. We focused on the main integration parameters: particle peak (Peak 1)-related LS90, A260, A280, TRP fluorescence, LS90, sizing, and immunolabelling result. A summary of the evaluated parameters is presented in Table 4.

As evident from the results in Table 4, the different tested conditions had minimal impact on light absorbance and tryptophan fluorescence parameters. Higher salt concentrations resulted in reduced Peak 1-related immunofluorescence. In relation to the preserved total antibody-related fluorescence, this indicates a reduced immunolabelling efficiency. The level of impact was different for different probes. Among the tested probes, the highest sensitivity to salt was observed for CD81 (over 30% decrease), followed by CD9 (approximately 10% decrease), while for CD63, the relative decrease (up to 5%) was smaller than the relative standard deviation of the reference sample in dPBS (6%). The possible pH impact on immunolabelling was within the method error for the tested set. Another major effect of preincubation in a high salt buffer was some increase in LS intensity with markedly larger variation (below 5% in low salt buffers, compared with 15% or above in the case of preincubation in high salt buffers), which was linked with a decrease in particle size. In the case of particle decomposition, one expects a decrease in LS parameters, coupled with a decrease and/or redistribution of absorbance, TRP fluorescence, and immunofluorescence among peaks with longer retention times. In the case of particle aggregation, increased LS intensity is expected to be associated with increased particle size. For these samples, the Peak 1 intrinsic tryptophan fluorescence and CD63 immunofluorescence were largely preserved. Therefore, the change in particle size and scattering intensity was interpreted as particle shrinkage due to the hypertonic conditions rather than particle degradation. These results are in line with the observations of the impact of salt concentration in the SEC running buffer on light scattering intensity described in the previous section (Section 2.2.2) and with observations described in a previous study [24].

This example demonstrates how multiparametric assessment is indispensable in studying sample integrity. The fact that the described effects could have been observed in the particle peak, which is expected to be completely buffer exchanged into the SEC mobile phase buffer before reaching detectors, indicates the sensitivity of the method even for some transient changes. Different characterisation parameters are unequally affected by different factors. To assure reliable results, evaluation parameters should be defined in advance, and sample preparation correspondingly adjusted to minimise undesired impacts. In the case of the introduction of new evaluation parameters, suitable controls should be implemented to validate the robustness of the analysis.

## 3. Discussion

Although significant advancements were made in the abilities of different established techniques for EV characterisation, most of them still rely on a relatively narrow dynamic range and often require some prior knowledge about the sample. In EV production and purification monitoring, a comprehensive characterisation of samples is required at a reasonable throughput. Few methods have been validated in the EV field that readily provide qualitative and quantitative information on EVs and impurities at the same time. Besides the analytical SEC considered in this study, asymmetrical flow field flow fractionation (AF4) with multidetector monitoring can be applied in a similar way [26]. When compared with SEC, AF4 is applicable to a wider particle size range, may provide higher resolution, and may be gentler to fragile particles in terms of degradation or aggregation [26]. Successful characterisation of EV samples using AF4 was demonstrated in several previous studies [27,28,29,30]. However, AF4 is likely to be more expensive and require a higher level of operator expertise for method development and adaptation. In Table 5, we summarise some of the pros and cons of the established EV characterisation techniques relevant for their applicability in process development.

In this study, we demonstrate the potential of SEC as a relatively fast and simple alternative to traditional multi-method evaluation of EV samples with exemplary evaluation of samples from two unrelated TFF processes. In this context, the SEC stands out with a significant advantage; the exploitation of general features of biological materials (light absorbance, scattering and fluorescence) and a rather wide dynamic range of detectors allows for the analysis of a wide range of samples with little prior knowledge on their composition and concentration (for orientation ranges applying to parameters in a representative EV sample, see Section 2.2.1 and Figure 3). This enables analysis of relatively EV-poor samples, such as culture conditioned media, and reduces the number of experiments required to adjust the sample dilution to target the method-suited concentration range. Moreover, the method provides information on the residual of non-particle-associated components. Quality assessment derived through evaluation of signal ratios and quantitation of non-particle-associated components can be used as a control of sample purity and integrity. These insights are crucial for reliable EV production monitoring, for better-directed purification processes, as also for efficient product control for further applications.

In our study, the rationale behind the selection of a silica-based column was the low level of expected non-specific interactions, compatibility with a physiological mobile phase, and a high-resolution chromatography system. We chose the column with a medium pore size, in which all particles larger than approximately 30 nm in size elute nearly together, close to the column void volume. The fact that all the nanoparticles elute closely adjacent allowed us to decrease the limit of detection compared with what could be achieved by further resolving particles on columns with larger pores. Furthermore, this type of column provided very efficient separation of the bound antibody from the probe excess.

The essence of the impact of the mobile phase on SEC separation is seldom discussed in the EV field, and the appropriateness of protocols developed for the separation of samples at physiological conditions is often taken for granted. However, the sample medium and the SEC mobile phase can be expected to influence the analysis result by affecting interactions among the sample components and between the sample components and the column solid phase, influencing the labelling efficiency and specificity, and impacting the intensity of signals (absorbance, fluorescence, and light scattering). In the present study, we tested the pH range between 6 and 8, salt concentration in the range of 0–300 mM, and the addition of poloxamer 188 as a widely used non-sticking and shear-preventing agent. Within the tested conditions, we observed little changes that could be unambiguously related to sample degradation, such as marked changes in proportions between the signals, redistribution of particle peak-related signals among the components eluting at longer elution times, or complete distortion of the profile. On the other hand, the impacts on sample separation and signal detection were significant, as detailed in Section 2.

The outcomes presented in Section 2.2 demonstrate that with respect to some critical parameters (e.g., sufficient incubation time, impacts of salt concentration on immunolabelling efficiency and particle shape, etc.), highly repeatable results can be obtained. The data suggest that the proposed approach is relatively robust to slight changes within a reasonable condition range. On the one hand, this assures that slight deviations in sample and buffer preparation should not substantially affect the analysis results; however, on the other hand, it allows for suitable adjustment of conditions if beneficial based on the focus of the analytics (e.g., due to the properties of a specific type of sample, applied dyes, fluorochromes, established buffers in the workflow, etc.).

Surely, no method is universal, and the limits should be borne in mind to prevent misinterpretations. One of the limitations of the described method is that although crude samples, such as conditioned media, can be analysed, some level of sample clarification is still required; for example, cells and larger cell debris should be removed with centrifugation or filtration through at least a 1-micron pore-sized filter to prevent column clogging.

Furthermore, the path to efficient and reproducible labelling is often slippery. Fluorescent chemical staining or immunlabeling is widely used to distinguish EVs from other particles. Although inappropriate protocols can generate confusing or inaccurate results, publications evaluating critical factors for equilibrium staining of EVs with different probes are rather rare. Immunolabelling optimisation usually aims to define two probe concentration limits: the maximal antibody concentration that minimally increases the background/noise and the minimal antibody concentration that allows for sensitive and reliable detection of positive events. Preferably, probe concentrations are set to achieve antigen-binding saturation. In many studies, the binding equilibrium after 1–2 h of incubation with the probe is taken for granted. As evident from the time dependency of the readout signal for an EV sample and three different probes presented in Figure 3c, the equilibrium establishment may often require much longer incubation times. Incubation times to equilibrium beyond 10 h are not unexpected for high-affinity probes [39]. Time to equilibrium depends on the antibody concentration, accessibility of the binding site, and probe affinity. Incubation temperature may also impact the dynamics; however, a higher temperature may not always increase the association rate [39] or assay readout [40]. Furthermore, the binding constants for the same antibody–epitope pairs may differ for samples of different origin (different particle types carrying the antigen, varying cell types, donors, etc.). Nevertheless, it can be expected that association-rate-related differences are decreased at longer incubation times, as previously described in the study of Tertel et al. [40].

As follows, equilibrium binding state assuring times extend with lower association rate and higher interaction affinity, and can become impractically long. Using higher probe concentrations may reduce the necessary incubation times, but would also increase the economic and ecological footprint of the analysis. When sufficient incubation times are infeasible, the time dependency model function can be built on a reference sample, and an assay-specific correction factor could be implemented.

Respecting the suitable equilibration time, consistent immunostaining results were obtained in samples from clarified conditioned medium to the TFF-enriched sample in our study. The results illustrate that immunolabelling dynamics differ significantly for different probes and that final labelling efficiency is not directly proportional to the number of present epitopes. The concentration of the probe can also be used to decrease the limit of detection for certain probes (see the results in Section 2.2.1), but reaching the binding saturation is not required to achieve a linear response. This is in contrast with some other immunolabelling-based techniques. In single particle analysis, such as flow cytometry, for example, the binding saturation is essential to ensure efficient detection of labelled particles, reliable separation between marker-positive and -negative populations, and reproducible characterisation [41]. As with any immunolabelling method, the probes should be suitably evaluated before implementation. The advantage of SEC compared with several other methods exploiting immunolabelling (e.g., flow cytometry or fluorescent-NTA) is that the probe concentration can be increased much more, as the excess probe is separated from the bound before detection, avoiding the impact of excess probe on increasing background.

Throughout EV downstream processing, samples may be introduced into the media that deviate from the physiological conditions. When analysing such in-process samples, it is advised that the impacts of sample matrices on labelling efficiency are tested. This can easily be carried out with a reasonably concentrated reference sample that can be spiked in different relevant media prior to adding the fluorescent probe.

The presented method can be easily adapted for a specific focus. We suggest that, when possible, samples that are to be directly compared are analysed within the same series, and that suitable controls are included within the sequence, e.g., sample stability control (reference sample spiked into the relevant media applied in the downstream process), labelling controls (incubation time control – a reference sample analysed at the beginning and end of a sample series; media impact control – a reference sample spiked into the relevant media before labelling). Suitable standards should be implemented in the assay for reliable quantitation.

## 4. Materials and Methods

### 4.1. Samples

Human bone marrow-derived mesenchymal stem cells (MSCs, ATCC, Manassas, VA, USA, Cat. PCS-500-012) were cultivated in growth media containing MSC NutriStem XF medium with a supplement mix (Sartorius, Beit Haemek, Galilee, Israel, Cat. 05-202-1A and 05-201-1U) and 2.5% human platelet lysate (hPL, PL BioScience, Aachen, Germany, Cat. PE21012). EV production was performed on the Ambr250 Modular system (Sartorius) using single impeller unbaffled vessels (Sartorius, Surrey, UK, Cat. 001-2A33). MSCs were seeded on SoloHill FACT III microcarriers (Sartorius, Ann Arbor, MI, USA, Cat. F-221-050) in growth media with 1% hPL and stirred intermittently for the initial attachment phase. After 4 h, additional hPL was added to reach a final concentration of 2.5%, and stirring was set to 100 rpm for the duration of the process. The pH was kept at 7.4, and the dissolved oxygen concentration was maintained at 40%. After a 4-day growth phase, microcarriers were allowed to settle by gravity, and the media was exchanged for EV production media composed of DMEM (Gibco, London, UK), Cat. A14430-01) with 1 g/L glucose (Gibco, New York, NY, USA, Cat. A24940-01), 2 mM GlutaMAX (Gibco, London, UK, Cat. 35050-038), sodium pyruvate (Gibco, New York, NY, USA, Cat. 11360-070), 0.4 g/L AlbuMAX II (Gibco, Auckland, New Zealand, Cat. 11021-029), 1% MEM non-essential amino acids (Gibco, New York, NY, USA, Cat. 11140-050), 1% ITS-X (Gibco, New York, NY, USA, Cat. 51500-056), 2.5 mg/L ascorbic acid (Sigma Aldrich, Hamburg, Germany, Cat. 49752), 1 mg/L reduced L-glutathione (Sigma Aldrich, Shanghai, China, Cat. G6013), and 0.1% trace elements B (Corning, New York, NY, USA Cat. 25-022-CI). After a 3-day production phase, conditioned media were collected from the vessels, passed through a 70 μm cell strainer (Miltenyi Biotec, Bergisch Gladbach, Germany, Cat. 130-098-462), and filtered through a Sartopure PP3 1.2 μm capsule filter (Sartorius, Göttingen, Germany, Cat. 5051303P4--SS). The samples were stored at −80 °C until further processing.

Clarified conditioned media (CCM) was then processed using tangential flow filtration (TFF) using a lab-scale TFF system (Repligen, Waltham, MA, USA, KR2i) and a 500 kDa cut-off hollow fibre membrane (Repligen, Waltham, MA, USA, Cat. D02 E500 05 N), keeping flux between 10 and 15 LMH and a trans membrane pressure of 1.5 psi. In the first step, CCM samples were concentrated approximately 10-fold (Process 1) or 5-fold (Process 2), followed by buffer exchange of the retentate (RET) for 5 diavolumes using commercial Dulbecco’s phosphate-buffered saline (dPBS, Biowest, Nuaillé, France, Cat. L0615-1000) in the case of “Process 1” or 30 mM Bis-Tris Propane (BTP), 50 mM NaCl, and 2% sorbitol at pH 7.25 in the case of “Process 2”. The retentate in Process 2 was further concentrated approximately 6-fold to obtain the final TFF RET sample. Permeate 1 was collected during the concentration phase, and permeate 2 was collected during diafiltration (only for Process 2). The system was flushed to recover all EVs with the “Flush” sample analysed separately for Process 2.

### 4.2. Chromatography

Chromatography was performed using the PATfix^®^ system (Sartorius, Ajdovščina, Slovenia, Cat. PAT0021), equipped with a conductivity and pH metre, a UV detector with a 50 mm optical path length flow cell cartridge, a fluorescence detector (Sartorius, Ajdovščina, Slovenia, Cat.PAT0023), and MALS detector (Sartorius, Ajdovščina, Slovenia, Cat. PAT0033). Absorbance was monitored at 260 and 280 nm. FITC fluorescence was excited at 488 nm, and emitted light was measured at 520 nm. Tryptophan fluorescence was excited at 280 nm, and emitted light was measured at 348 nm. Light scattering was monitored at 9 angles (28°, 44°, 76°, 60°, 90°, 108°, 124°, 140°, and 156°). Polystyrene size standard beads (Postnova, Landsberg, Germany, Cat. Z-PS-POS-000-0,04; Z-PS-POS-000-0,06; Z-PS-POS-000-0,1) were used for MALS signal normalisation and/or sizing control. Normalised intensities were fitted to software-integrated theoretical curves for particle size recalculation. Only fits with R^2^ exceeding 0.98 were considered relevant. To provide more intuitive concentration-related sample characteristics, the integrated peak area below the different signal response curves was multiplied by the sample dilution factor and divided by the sample injection volume and expressed as relative units (relative fluorescence units (RFU)/relative absorbance units (RAU)/relative scattering units (RSU)) per millilitre.

Separation was performed on a TSKgel G4000SWXL Column (Tosoh Bioscience, Tokyo, Japan) at a flow rate of 1 mL/min. A 30 min method was applied to prevent carry over between the samples. The runtime was shortened to 18 min for purified samples after confirming absence of components with extended retention times. Data processing and evaluation of chromatograms were carried out with PATfix™ software versions 2.1 and 4.0 (Sartorius, Slovenia). In immunolabelled samples, the integrated area of fluorescence signal was used to evaluate particle-bound and unbound probes. Immunolabelling was carried out with FITC-conjugated antibodies targeting human CD9, CD63, and CD81. Antibodies were purchased from Biolegend, San Diego, USA, (CD9: Cat. 312104; CD63: Cat. 353006; CD81: Cat. 349504; and IgG isotype control: Cat. 400110). BEH200 SEC Test Mix (Waters, USA, Cat. 186006518) was prepared following the manufacturer’s instructions and used to control column integrity and as a protein size standard. Absorbance response curve at 280 nm for protein evaluation was derived using bovine serum albumin (BSA) standard (Thermo Scientific, Waltham, USA, Cat. 23209).

### 4.3. Nanoparticle Tracking Analysis

Nanoparticle tracking analysis (NTA) was performed using NanoSight^®^ NS300 (Malvern Panalytical Inc., Malvern, UK), employing a blue laser with a wavelength of 488 nm. Samples were diluted in dPBS to reach a concentration of particles between 20 and 80 particles/frame. Five videos of 60 s were recorded for each sample. Measurement was performed at 25 °C, and the syringe pump speed was set to 25. The video images were analysed with the system-incorporated Nanosight NTA 3.2 software. The size mode (nm) and concentration (particle number/mL) of the EVs were calculated by combining the data from the five records.

### 4.4. Protein Analysis

The Pierce™ Coomassie Plus (Bradford) Assay Kit (Thermo Scientific, Waltham, MA, USA, Cat. 23236) was used according to the manufacturer’s instructions to evaluate the protein contents of the samples. Light absorbance at a wavelength of 595 nm was measured using a Synergy H1 Hybrid Microplate Reader. Recalculation of protein concentration was based on a standard curve obtained with BSA (Thermo Scientific, Waltham, MA, USA, Cat. 23209) (details as Appendix A Figure A1).

### 4.5. Design of Experiments for Method Optimisation and Identification of Critical Parameters for Repeatable Assay Readout

#### 4.5.1. Sample and Antibody Titration

TFF-retentate (TFF-RET) was diluted in dPBS to reach final concentrations of 5 × 10^8^, 1 × 10^9^, 5 × 10^9^, and 1 × 10^10^ particles/mL (according to NTA). Different amounts of antibody probes were added to the sample to reach the final concentrations of probes in the samples: 50, 125, 250, 625, and 1250 ng/mL. A matrix of samples was prepared on a microtiter plate as illustrated in Table 6. After preparation, the samples were mixed gently for 1 h at room temperature in the dark. Then, the microtiter plate was transferred into the autosampler, which was pre-chilled to 4 °C. The samples were incubated in the dark in the autosampler until injection, for at least 12 h. The running buffer composition for titration experiments consisted of 50 mM MES, 150 mM NaCl, and 0.05% poloxamer, at pH 6.5.

#### 4.5.2. Evaluation of the Impact of Incubation Time

The TFF-RET sample was diluted in dPBS to the target concentration of 2.5 × 10^9^ particles/mL. Antibody probes were added at a final concentration of 625 ng/mL. The samples with added probes were vortexed, dispensed in several aliquots on a microtiter plate, and then immediately placed into the autosampler, which was pre-chilled to 4 °C. Aliquots were injected after different incubation times, from 0.5 h up to 48 h. The running buffer composition for titration experiments consisted of 50 mM MES, 150 mM NaCl, and 0.05% poloxamer, at pH 6.5.

#### 4.5.3. Evaluation of the Impact of Running Buffer Composition

2-(N-morpholino)ethanesulfonic acid (MES, Sigma Aldrich, Saint Louis, MO, USA, Cat. 69892), 4-(2-hydroxyethyl)-1-piperazineethanesulfonic acid (HEPES, Sigma Aldrich, Saint Louis, MO, USA, Cat. 1.10110.1000), KH_2_PO_4_ (Merck, Darmstadt, Germany, Cat. 1.04873.1000), K_2_HPO_4_ (Merck, Darmstadt, Germany, Cat. 1.05104.1000), NaCl (Merck, Darmstadt, Germany, Cat. 1.06404.1000), and Poloxamer 188 (Merck, Rahway, NJ, USA, Cat. 1.37065.9012) were used to prepare buffer stock solutions as listed in Appendix A Table A1. Various SEC mobile phases were prepared inline using a PATfix-incorporated quaternary pump, mixing stock solutions in selected proportions. The final conditions consisted of 25 mM buffer (HEPES/MES/Phosphate) with pH values ranging from 5.5 to 8 (each buffer was used in its corresponding buffering range), salt concentration between 0 mM NaCl and 300 mM NaCl, and poloxamer 188 additive between 0% and 0.1%. The list conditions applied is provided in the Appendix A Table A2. The final running buffer pH and conductivity were controlled by PATfix inline detectors. The collected data was statistically evaluated using MODDE software (Sartorius, Sweden), version 13.

#### 4.5.4. Evaluation of the Impacts of Salt and pH on Immunolabelling Efficiency

TFF RET was 10-fold diluted in different buffers (listed in Table 7) before adding the antibody probe solution (final probe concentration of 625 ng/mL). The pH and salt ranges were chosen based on the EV literature [21,24,42]. The resulting chromatograms were compared to evaluate the influence of the sample matrix on particle profile and on immunolabelling efficiency.

## Figures and Tables

**Table 1:** The results of the characterisation of samples from two TFF processes.

		V [mL]	NTA Particle Conc. [Particles/mL]	LS90 [RSU/mL]	NTA Sizing Mode, Mean (D10-D90) [nm]	MALS Sizing at Peak Apex (Range ^a^) [nm]	Protein Conc. Bradford Assay [µg/mL]	Protein Conc. Estim. ^b^ from SEC A280 [µg/mL]	Protein Depletion Bradford Assay	Protein Depletion Estim. ^b^ (SEC A280)	CD9 [RFU/mL]	CD63 [RFU/mL]	CD81 [RFU/mL]
Process 1	CCM	200	2.30 × 10^9^	15 ± 1	145 ± 7, 170 ± 1 (112–236)	169 ± 12 (126–252)	527 ± 20	523 ± 36	n.a.	n.a.	11.6	24.6	13.5
	PER1	180	n.a.	n.a.	n.a.	n.a.	213 ± 5	252 ± 13	n.a.	n.a.	bld	bld	bld
	RET+Flush	20	1.20 × 10^10^	67 ± 3	140 ± 7, 172 ± 1 (119–241)	171 ± 7 (120–242)	251 ± 8	201 ± 22	−95%	−96%	132.5	283.3	137.6
Enrichment factor			5	4	Enrichment factor						11.4	11.5	10.2
Process 2	CCM	400	1.16 × 10^9^	13 ± 2	130 ± 2, 192 ± 2 (122–292)	194 ± 12 (152–284)	479 ± 55	504 ± 2	n.a.	n.a.	20.00	34.9	18.3
	PER1	320	n.a.	n.a.	n.a.	n.a.	258 ± 52	267 ± 8	n.a.	n.a.	bld	bld	bld
	PER2	620	n.a.	n.a.	n.a.	n.a.	191 ± 47	84 ± 10	n.a.	n.a.	bld	bld	bld
	RET	12	2.99 × 10^10^	271 ± 40	161 ± 8, 215 ± 3 (136–327)	183 ± 9 (165–248)	3405 ± 73	3019 ± 119	−79%	−82%	486.8	924.2	536.3
	Flush	15	5.17 × 10^9^	35 ± 1	157 ± 6, 211 ± 5 (132–312)	188 ± 2 (151–287)	309 ± 38	359 ± 4	n.a.	n.a.	48.3	97.7	62.5
Enrichment factor			26	21	Enrichment factor						24.3	26.5	29.2

SEC analytics-extracted parameters are marked with a grey background. The data obtained with orthogonal methods is provided in cells with a white background. Enrichment factors correspond to the relative increase in the parameter in TFF retentate (RET + Flush/RET) compared with the starting material (CCM). n.a.—not applicable; bld—below the limit of detection; RSU—relative scattering units; RFU—relative fluorescence units. ^a^ The high and the low values of the range were obtained at the beginning and the end of the peak, respectively. Size determination is only possible where signal intensity and quality are sufficient, which may vary from sample to sample, with concentration and polydispersity. ^b^ Protein concentration estimation is based on light absorbance at 280 nm as read from the SEC chromatogram. Recalculation was based on the BSA standard curve. The evaluation of possible interferences of other sample components (e.g., nucleic acids) was beyond the scope of this study.

**Table 2:** Correlation matrix for selected parameters in the SEC evaluation of the protein mix.

		Factors						Responses	
		Buf(B)	Buf(C)	pH	c NaCl	Cond.	% *p*188	RT Pk1	Res 2–3
Factors	Buf(B)	1	0.41	0.28	−0.09	−0.19	0.08	−0.34	−0.25
	Buf(C)	0.41	1	−0.59	−0.09	−0.19	0.07	0.17	0.22
	pH	0.28	−0.59	1	0.00	0.05	−0.02	−0.30	−0.39
	c NaCl	−0.09	−0.09	0.00	1	0.99	0.30	0.51	0.70
	Cond.	−0.19	−0.19	0.05	0.99	1	0.28	0.54	0.69
	% *p*188	0.08	0.07	−0.02	0.30	0.28	1	0.17	0.20
Resp.	RT Pk 1	−0.34	0.17	−0.30	0.51	0.54	0.17	1	0.75
	Res 2–3	−0.25	0.22	−0.39	0.70	0.69	0.20	0.75	1

Buf(A/B/C)—buffer type (A—potassium phosphate/B—HEPES/C—MES). Cond.—buffer conductivity. % *p*188—% poloxamer 188. RT Pk1—retention time of protein Peak 1 (largest protein in the mix). Res 2–3—resolution between Peaks 2 and 3 (the least-resolved protein peaks in the mix). Repeated correlation coefficient values are written in grey.

**Table 3:** Correlation matrix for selected parameters evaluated in SEC chromatograms of the EV sample.

		Factors			Responses										
		pH	c NaCl	% *p*188	FLD RT Pk1	FLD Area Total	FLD Res 1–2	FLD Res 2–3	FLD S/T Pk1	FLD S/T Pk2	FLD S/T Pk3	LS90 Pk1 RT	Rel. LS90 Pk1 Area	Rel. LS Pk1 Height	LS90 S/T
Factors	pH	1	−0.07	−0.08	−0.16	0.98	0.35	0.81	−0.20	−0.81	−0.25	−0.16	0.41	0.44	−0.53
	c NaCl	−0.07	1	0.30	0.36	0.02	0.32	−0.01	−0.46	−0.05	−0.29	−0.16	0.34	0.52	0.25
	% *p*188	−0.08	0.30	1	0.36	−0.02	−0.04	−0.11	−0.18	0.23	−0.15	−0.07	0.12	0.17	0.19
Responses	FLD RT Pk1	−0.16	0.36	0.36	1	−0.08	0.41	−0.29	−0.07	0.02	−0.03	0.34	−0.20	0.01	0.27
	FLD RT Pk2	−0.60	0.41	0.16	0.69	−0.51	0.41	−0.64	−0.08	0.26	0.29	0.39	−0.44	−0.30	0.59
	FLD RT Pk3	−0.81	0.38	0.15	0.52	−0.73	0.01	−0.65	−0.03	0.62	0.06	0.35	−0.39	−0.36	0.60
	FLD W05 Pk1	−0.57	0.26	−0.12	0.00	−0.55	−0.11	−0.52	−0.23	0.46	0.12	−0.04	−0.23	−0.36	0.43
	FLD W05 Pk2	−0.80	−0.10	0.17	0.00	−0.84	−0.79	−0.77	0.17	0.95	−0.11	−0.01	−0.13	−0.27	0.23
	FLD W05 Pk3	−0.74	0.28	0.04	0.45	−0.73	−0.03	−0.77	−0.05	0.57	0.07	0.31	−0.35	−0.37	0.53
	FLD Area Total	0.98	0.02	−0.02	−0.08	1	0.44	0.84	−0.25	−0.82	−0.26	−0.14	0.40	0.46	−0.49
	FLD % Area Pk1	0.23	0.44	0.01	0.08	0.21	0.29	0.04	−0.67	−0.20	−0.18	−0.31	0.12	0.14	0.21
	FLD % Area Pk2	0.26	−0.23	0.07	0.01	0.29	−0.26	0.43	0.36	0.01	−0.47	0.30	0.14	0.14	−0.43
	FLD % Area Pk3	−0.57	−0.19	−0.11	−0.10	−0.59	0.02	−0.57	0.28	0.20	0.78	−0.03	−0.30	−0.32	0.31
	FLD Res 1–2	0.35	0.32	−0.04	0.41	0.44	1	0.30	−0.19	−0.73	0.35	0.21	−0.13	0.10	0.15
	FLD Res 2–3	0.81	−0.01	−0.11	−0.29	0.84	0.30	1	−0.14	−0.68	−0.25	−0.15	0.34	0.36	−0.40
	FLD S/T Pk1	−0.20	−0.46	−0.18	−0.07	−0.25	−0.19	−0.14	1	0.09	0.27	0.36	−0.48	−0.42	−0.03
	FLD S/T Pk2	−0.81	−0.05	0.23	0.02	−0.82	−0.73	−0.68	0.09	1	−0.16	0.04	−0.16	−0.31	0.29
	FLD S/T Pk3	−0.25	−0.29	−0.15	−0.03	−0.26	0.35	−0.25	0.27	−0.16	1	0.18	−0.43	−0.42	0.24
	LS90 RT	−0.16	−0.16	−0.07	0.34	−0.14	0.21	−0.15	0.36	0.04	0.18	1	−0.49	−0.49	0.22
	Rel. LS90 Pk1 Area	0.41	0.34	0.12	−0.20	0.40	−0.13	0.34	−0.48	−0.16	−0.43	−0.49	1	0.90	−0.54
	Rel. LS Pk1 Height	0.44	0.52	0.17	0.01	0.46	0.10	0.36	−0.42	−0.31	−0.42	−0.49	0.90	1	−0.49
	LS90 W05	−0.36	−0.25	−0.16	−0.22	−0.38	−0.13	−0.28	0.08	0.31	0.27	0.40	−0.52	−0.75	0.44
	LS90 S/T	−0.53	0.25	0.19	0.27	−0.49	0.15	−0.40	−0.03	0.29	0.24	0.22	−0.54	−0.49	1

% *p*188—% poloxamer 188. Pk1—Peak 1, particle peak. Pk2—Peak 2, unbound antibody probe. Pk3—Peak 3, free fluorochrome. RT—retention time. Res 1–2—resolution between Peaks 1 and 2. S/T—symmetry/tailing. W05—mean peak width. FLD—fluorescence. LS90—light scattering at 90° angle. rel.—relative value. The cell background is shaded from white to dark green to highlight the strength of correlations, with darker shades indicating stronger correlations. Repeated correlation coefficient values are written in grey.

**Table 4:** The result of SEC characterisation of the EV sample spiked into 7 different buffers.

	**A280 [RAU/mL]**	**rΔA280** **%**		**TRP** **(Peak 1)** **[RFUs/mL]**		**rΔTRP (Peak 1)** **%**		**TRP** **(Total)** **[RFUs/mL]**	**rΔTRP** **%**		**LS90** **[RSUs/mL]**		**rsd LS90** **%**	**rΔLS90** **%**	**MALS Size [nm]**		**rsd Size** **%**		**rΔSize** **%**
50 mM NaCl, pH 6.5	508	−3%		14,346		+1%		278,361	+1%		474		3%	−4%	105		4%		+2%
50 mM NaCl, pH 7.2	528	0%		14,418		+2%		283,596	+3%		515		2%	+4%	101		3%		−2%
50 mM NaCl, pH 8.0	534	+2%		15,096		+7%		288,651	+5%		515		3%	+4%	97		2%		−5%
1 M NaCl, pH 6.5	530	+1%		14,576		+3%		300,365	**+9%**		641		20%	**+30%**	64		5%		**−38%**
1 M NaCl, pH 7.2	534	+1%		14,484		+2%		299,127	**+9%**		592		15%	**+20%**	66		4%		**−36%**
1 M NaCl, pH 8.0	543	+3%		14,508		+3%		300,481	**+9%**		595		15%	**+20%**	64		6%		**−38%**
137 mM NaCl, pH 7.4 (dPBS reference)	526	n.a.		14,134		n.a.		275,244	n.a.		494		8%	n.a.	103		4%		n.a.
	**CD9** **(Peak 1)** **[RFUs/mL]**		**rΔCD9** **%**		**CD9** **(Total) [RFUs/mL]**		**CD63** **(Peak 1)** **[RFUs/mL]**			**rΔCD63** **%**		**CD63** **(Total) [RFUs/mL]**		**CD81** **(Peak 1)** **[RFUs/mL]**		**rΔCD81** **%**		**CD81** **(Total) [RFUs/mL]**	
50 mM NaCl, pH 6.5	513		+2%		13,351		910			±0%		10,141		576		+1%		14,222	
50 mM NaCl, pH 7.2	487		−3%		13,775		928			+2%		10,289		611		+7%		14,905	
50 mM NaCl, pH 8.0	506		+1%		13,820		926			+2%		10,234		602		+6%		14,786	
1 M NaCl, pH 6.5	460		**−8%**		13,671		877			−3%		10,127		379		**−33%**		14,454	
1 M NaCl, pH 7.2	451		**−10%**		13,603		868			−4%		10,230		390		**−31%**		14,836	
1 M NaCl, pH 8.0	451		**−10%**		13,675		860			−5%		10,143		363		**−36%**		14,497	
137 mM NaCl, pH 7.4 (dPBS reference)	502		(±3%)		13,283		907			(±6%)		9731		569		(±1%)		14,363	

A280—light absorbance at 280 nm. TRP—intrinsic tryptophan fluorescence. CD9—CD9-related immunofluorescence. CD63—CD63-related immunofluorescence. CD81—CD81-related immunofluorescence. LS90—light scattering at 90° angle. MALS size—multi-angle light scattering-derived size. rΔ—relative difference in the measured parameter compared with the reference sample; note that for the CD markers in the dPBS sample, the rsd of replicates is shown (in brackets) instead of the relative difference. Relative difference values exceeding the expected variation due to the method error are marked in bold. Peak 1—particle peak. rsd—relative standard deviation.

**Table 5:** Comparison of the applicability of some common EV characterisation techniques in process monitoring.

Technique	Output Information				Benefits	Limitations	Ref.
	Particle Concentration	Particle Size/Distribution	Marker Profile	Impurity Profile			
TRPS	+	+	−	−	Simple	Limited throughput; might not be suitable for complex samples; requires prior estimation of particle concentration	[31,32,33]
NTA	+	+	−/+ ^a^	−	Simple	Presence of larger particles may impair analysis; fluorescence detection is prone to bleaching effects; evaluation of fluorescent particles is limited by the number of marker molecules per particle and fluorochrome brightness; fluorescent labelling requires extensive optimisation, often including additional sample pretreatment to remove excess fluorescent probe; requires prior estimation of particle concentration	[32,34]
FCM	+	−/+ ^b^	+	−	Applicable to samples with low particle concentration and highly customisable	Hardly encounters the complete population of EVs/nanoparticles due to the limited sensitivity of scattering and fluorescence detectors; small changes in the lower limit of detection can lead to great differences in measured EV concentrations; fluorescent labelling requires extensive optimisation, often including additional sample pretreatment to remove excess fluorescent probe; requires prior estimation of particle concentration	[34,35,36,37]
Commercial single particle imaging platforms (e.g., ExoView)	+ ^c^	+ ^c^	+	−	Easy to perform, automated, and applicable to complex samples	Only particles carrying specific markers are encountered in evaluation; requires prior estimation of particle concentration	[38]
AF4 (suppl. with UV, LS, and FLD detectors)	Relative (LS-intensity-based)	+	+	+	High resolution, gentle to fragile particles, highly customisable, and applicable to complex samples	Expensive; requires a rather concentrated sample; method development and optimisation require special expertise; LS-intensity-weighted size distribution	[26,27,28,29]
Analytical SEC (suppl. with UV, LS, and FLD detectors)	Relative (LS-intensity-based)	+	+	+	Applicable to complex samples; customisable; the sensitivity for marker detection increased due to the cumulative signal across the population	Does not provide number-based particle concentrations; sensitivity for nanoparticles at the expense of limited resolution; LS-intensity-weighted size distribution	

^a^ Requires fluorescence detection module (fNTA). ^b^ Requires additional data treatment. ^c^ Only marker-positive particles included in evaluation.

**Table 6:** Titration matrix.

		Antibody Concentration (ng/mL)					
		1250	625	250	125	50	0 (No Add. Control)
Sample conc. (particles/mL)	1 × 10^10^	A1	A2	A3	A4	A5	A6
	5 × 10^9^	B1	B2	B3	B4	B5	B6
	1 × 10^9^	C1	C2	C3	C4	C5	C6
	5 × 10^8^	D1	D2	D3	D4	D5	D6
	0 (Antibody Control)	E1	E2	E3	E4	E5	Blank

**Table 7:** List of conditions to analyse the impact of the sample medium on the SEC analysis result.

Condition Description	Buffer Type	Buffer	NaCl Concentration	pH
Physiological	dPBS	9 mM phosphate	137 mM	7.4
Low salt, low pH	MES	50 mM MES	50 mM	6.5
Low salt, mid pH	HEPES	50 mM HEPES	50 mM	7.2
Low salt, high pH	HEPES	50 mM HEPES	50 mM	8.0
High salt, low pH	MES	50 mM MES	1 M	6.5
High salt, mid pH	HEPES	50 mM HEPES	1 M	7.2
High salt, high pH	HEPES	50 mM HEPES	1 M	8.0

## Data Availability

All data related to the conclusions are included within this manuscript and/or the Supplementary Materials. The raw datasets generated within the study are available from the corresponding author upon reasonable request.

## Supplementary Materials

The following supporting information can be downloaded at https://www.mdpi.com/article/10.3390/ijms26178477/s1.

## Abbreviations

The following abbreviations are used in this manuscript:

A260	Absorbance at a wavelength of 260 nm
A280	Absorbance at a wavelength of 280 nm
BSA	Bovine serum albumin
CCM	Clarified conditioned medium
dPBS	Dulbecco’s phosphate-buffered saline
EV	Extracellular vesicle
FLD	Fluorescence
FLD 280/348	Fluorescence signal using excitation at 280 nm and emission detection at 348 nm
LS	Light scattering
LS90	Light scattering at a 90° angle
MALS	Multi-angle light scattering
MSC	Mesenchymal stem cell
NTA	Nanoparticle tracking analysis
RAU	Relative absorbance units
RET	Retentate
RFU	Relative fluorescence units
RSU	Relative scattering units
SEC	Size-exclusion chromatography
TFF	Tangential flow filtration
TRP	Tryptophan autofluorescence

## Appendix A

The response curves for bovine serum albumin (BSA) for absorbance at 280 nm (A280) and fluorescence with excitation at 280 nm and emission at 348 nm (FLD 280/348).

**Table A1:** List of stock solutions for running buffer preparation.

	A	B	C	D
**1**	50 mM HEPES pH 6.8	50 mM HEPES pH 6.8, 0.2% poloxamer	600 mM NaCl	ddH_2_O
**2**	50 mM HEPES pH 8, 0.2% poloxamer	50 mM HEPES pH 8		
**3**	50 mM MES pH 5.5	50 mM MES pH 5.5, 0.2% poloxamer		
**4**	50 mM MES pH 6.5, 0.2% poloxamer	50 mM MES, pH 6.5		
**5**	50 mM KP, pH 6.4	50 mM KP, pH 6.4, 0.2% poloxamer		
**6**	50 mM KP pH 7.8, 0.2% poloxamer	50 mM KP, pH 7.8		
**7**	Same as B1	Same as A6		
**8**	Same as B3	dPBS		

**Table A2:** List of applied running buffer conditions.

No. ID	Buffer	A		B		C	D	NaCl, mM	Poloxamer %	pH	Cond.
**1, 41**	dPBS	-	0%	B8	100%	0%	0%	150	0	7.4	15.4
**2**	HEPES	A1	50%	-	0%	0%	50%	0	0	6.6	0.15
**3**	HEPES	A2	50%	-	0%	50%	0%	300	0.1	7.8	29.3
**4**	HEPES	A2	35%	B2	15%	8%	42%	48	0.07	7.9	6.2
**5**	HEPES	A2	16%	B2	34%	27%	23%	162	0.03	7.8	17.2
**6**	HEPES	-	0%	B2	50%	25%	25%	150	0	7.8	16.1
**7**	HEPES	A1	25%	B2	25%	50%	0%	300	0	6.8	26
**8**	HEPES	A7	25%	B2	25%	17%	33%	102	0.05	7.3	11.1
**9**	HEPES	A7	30%	B2	20%	28%	22%	168	0.06	7.2	17.5
**10**	HEPES	A7	25%	B2	25%	26%	24%	156	0.05	7.2	16.6
**11**	HEPES	A7	25%	B2	25%	32%	18%	192	0.05	7.2	20
**12**	HEPES	A2	25%	B1	25%	0%	50%	0	0.1	7.3	0.6
**13**	HEPES	A2	25%	B1	25%	22%	28%	132	0.1	7.3	13.8
**14**	HEPES	-	0%	B1	50%	25%	25%	150	0.1	6.6	15
**15**	HEPES	A1	25%	B1	25%	50%	0%	300	0.05	6.6	28.4
**16**	HEPES	A1	50%	-	0%	2%	48%	12	0	6.7	2
**17**	MES	A3	50%	-	0%	10%	40%	60	0	5.4	6.6
**18**	MES	A3	25%	B3	25%	50%	0%	300	0.05	5.4	28.4
**19**	MES	-	0%	B3	50%	25%	25%	150	0.1	5.3	15.1
**20**	MES	-	0%	B3	50%	42%	8%	252	0.1	5.3	24.5
**21**	MES	A4	25%	B3	25%	12%	38%	72	0.1	5.9	8.15
**22**	MES	A8	15%	B4	35%	21%	29%	126	0.03	6	13.4
**23**	MES	A8	30%	B4	20%	18%	32%	108	0.06	5.8	11.6
**24**	MES	A3	25%	B4	25%	35%	15%	210	0	5.9	21.4
**25**	MES	A3	50%	-	0%	25%	25%	150	0	5.7	15.7
**26**	MES	-	0%	B4	50%	23%	27%	138	0	6.3	15
**27**	MES	A4	20%	B4	30%	15%	35%	90	0	6.4	10.3
**28**	MES	A4	50%	-	0%	5%	45%	30	0	6.4	4.5
**29**	Phosphate	-	0%	B5	50%	21%	29%	126	0.1	6.6	14.7
**30**	Phosphate	A5	35%	B5	15%	4%	46%	24	0.03	6.5	5.5
**31**	Phosphate	A5	35%	B5	15%	45%	5%	270	0.03	6.2	28.2
**32**	Phosphate	A5	50%	-	0%	25%	25%	150	0	6.2	17.8
**33**	Phosphate	A5	25%	B6	25%	5%	45%	30	0	6.9	7
**34**	Phosphate	A5	25%	B7	25%	32%	18%	192	0.05	6.7	22.3
**35**	Phosphate	A6	25%	B5	25%	20%	30%	120	0.1	7	15.15
**36**	Phosphate	A6	50%	-	0%	35%	15%	210	0.1	7.3	24.2
**37**	Phosphate	A6	50%	-	0%	50%	0%	300	0.1	7.2	32
**38**	Phosphate	A6	10%	B6	40%	12%	38%	72	0.02	7.44	11.6
**39**	Phosphate	-	0%	B6	50%	28%	22%	168	0	7.3	21
**40**	Phosphate	A5	20%	B6	30%	40%	10%	240	0	6.8	26.8
